# Postgraduate radiology education: what has Covid-19 changed?

**DOI:** 10.1259/bjro.20200064

**Published:** 2021-07-22

**Authors:** Andrew Nanapragasam, Meghavi Mashar

**Affiliations:** 1 University Health Network, Toronto, Canada; 2 University College London Hospitals NHS Foundation Trust, London, United Kingdom

## Abstract

Radiology training in the UK follows a standardised pathway with formative and summative assessments throughout. The Covid-19 pandemic has affected multiple existing educational methods commonly used during radiology training including small group teaching, multidisciplinary team meetings, online e-learning modules, radiology courses, exam provision and more. As such, significant adaptations have been implemented in order to maintain the standard of radiology training which come with their respective advantages and disadvantages. However, the question still remains as to the effectiveness of these methods, their acceptability and longevity. In this review, we discuss these educational adaptations and future directions for training in the ongoing pandemic.

## Introduction

Radiology training in the UK follows a standardised pathway during which time registrars acquire the necessary competencies to practice independently. The programme begins with 3 years of core radiology training, followed by 2–3 years of subspeciality training.^1^

A descriptive imaging curriculum, made of an itemised list of competencies and skills, is used as the template for radiology education. Registrars undergo a series of workplace-based formative and summative assessments, which evidence the attainment of the various curriculum items, and these assessments are used to approve their progression to the next year of training. In addition, trainees must pass four nationally administered Fellow of the Royal College of Radiologists (FRCR) examinations in order to successfully qualify.

The COVID-19 pandemic has impaired the delivery of radiology training in a number of ways.^2^ Experiential learning has been compromised due to the partial suspension of outpatient/elective work and the reduced imaging case mix available for reporting.^3^ Some institutes have also had to cancel or postpone in-person teaching as social distancing could not be maintained.

We discuss the existing pedagogy in radiology education, present the challenges posed by the COVID-19 pandemic, describe accommodations that institutions and organisations have made to account for these challenges and how these adaptations may persist in the future

## Existing educational paradigms in radiology education

The learning during radiology training is primarily through an apprenticeship model,^4^ with iterations of feedback given on an individual basis, resulting in incremental improvement in the trainee’s performance. This one-to-one approach provides customised feedback, but the educational benefit is limited to a single trainee at a time.

Teaching is also delivered to groups of registrars, as it is more efficient to simultaneously teach multiple trainees, and because it provides educational equanimity across the cohort. Group teaching is often delivered in the form of daily/weekly local departmental teaching and is supplemented by monthly regional group teaching delivered to a larger audience of trainees.

Multidisciplinary team meetings (MDMs) are a rich learning environment,^4^ as they allow trainees to see a number of cases in quick succession. The learning is limited by not being able to independently scrutinise the images oneself during the MDMs, but the imaging can always be reviewed before or after the meeting. MDMs can be difficult to attend depending on other workplace commitments or limitations on room size.

With technological advances in recent years, e-learning methods such as online modules have become a part of imaging education. In the UK, the Radiology-Integrated Training Initiative (R-ITI) is a free online collection of modules^5^ that covers the majority of the radiology curriculum. The R-ITI modules provide both education for clinical competencies and exam specific content for the FRCR Part 1 Physics exam. The FRCR 2B examination assesses image interpretation through written answers and through a viva component.^4^ In order to maximise the chances of passing the 2B examination, candidates require dedicated practice with cases representative of those in the exam, and they require in person viva practice in order to rehearse and refine their presentation of findings. Typically, this is achieved through a combination of online image question banks and in person viva courses.

Although the fundamental principles of image interpretation and interventional radiology can be acquired in any programme, there is significant variation in the provision of certain subspecialities in some programmes. For trainees with an interest in attaining competencies that are not locally available, they may need to attend other training programmes. This can be achieved within the duration of the standard training programme, or after completion of training in the form of a fellowship.^6^

## Adaptations to radiology education due to COVID-19

The need for social distancing during the COVID-19 pandemic has caused significant interruption to radiology training, resulting in the need for innovative methods to continue teaching.

Traditionally group teaching sessions involve the convening of trainees from the department, or from across multiple departments, to a single site. The intermixing of trainees from different sites and the inability to socially distance once in the lecture room, both preclude this means of education during a pandemic. To solve this problem, departmental teaching has been moved online, using platforms like *Zoom*^7^ and *Microsoft Teams,* the latter being supported by NHS Digital.^8^

Although remote teaching was intended to avoid close contact of trainees, it has many other inadvertent benefits. Trainees who are not scheduled to be in the hospital at the time of the teaching session can still access it from their home. This is of benefit to those who would like to attend whilst self-isolating, on a rest days, or on maternity/paternity leave. Moreover, for those trainees who may be at other sites, it removes travel as a barrier to attending sessions.

Once a teaching session has been moved online, all physical barriers are removed, and the potential audience is practically limitless. As such, teaching sessions no longer have to be confined to a single hospital site or deanery. The Leeds Radiology Academy^9^ and the British Society of Neuroradiology^10^ in the UK, as well as the Emory Musculoskeletal Radiology^11^ department in the USA are examples of programmes that have freely shared access to their teaching sessions to trainees from across the world. Many of these sessions are available on-demand allowing trainees to access the recording after the initial broadcast.

This practice lessens the pre-existing duplication of education, as individual departments no longer need to provide the same teaching, as one centre can cover a topic for all trainees. Efficiency with teaching time is important during a pandemic as resources are often stretched and time for teaching is sacrificed in favour of clinical demands. Furthermore, centralised online teaching can allow the best speakers the largest platform, levelling the access to the highest quality educator. This is a tool being used by the London and South East School of Radiology, where all regional teaching is now being delivered online. With suitable permission, this may allow trainee collaboration across training schemes to share knowledge. For example, trainees in subspeciality centres elsewhere in the country may be able to share their teaching resources with ease.

Remote teaching additionally has other benefits for larger groups. Other advantages to online teaching are intrinsic to its format. Lectures and seminars have incorporated audience response techniques. These allow real-time polls to assess student’s knowledge and gauge their opinions, promoting interactivity and active learning. Furthermore, with remote small group teaching, this gives trainees the opportunity to experience the current format of the FRCR 2B examinations.

However, there are limitations associated with these teaching innovations. On an individual scale, it may restrict the opportunities junior trainees have to meet and interact with their consultants. As more and more consultants work from home, this may limit the exposure trainees have to various subspecialities and affect the working relationships between registrars and consultants. Moreover, virtual teaching poses challenges for the tutors. Many small group sessions involve attendees in the tutor keeping webcams off and repeatedly having to mute and unmute their microphones. Tutors thus cannot use facial expressions or body language to gauge understanding or how the session is being received. Issues with the microphone can lead to delayed or absent responses to questions, making interaction more challenging. For more complex concept which may necessitate diagrammatic representation or 3D models, these again may be more challenging to execute over virtual platforms.

Multidisciplinary meetings (MDMs) are another reason for people to gather in large groups. Just as with remote teaching, these meetings have also been moved online. Remote access to MDMs not only shares the same benefits listed above for remote teaching, but also has an additional benefit specific to radiology trainees. During the MDM, the radiologist will demonstrate the pathology on the screen and states the relevant findings in a manner that allows the medical and/or surgical teams to discuss treatment options. However, the radiologist will not typically demonstrate their thought process that allowed them to reach the conclusion they reached. As such, radiology trainees observing the meeting are only able to see the relevant final image, and not the radiological interpretive process. With remote access to MDMs, the radiology trainee observing the meeting can open the scan on their own computer and access the imaging in real time. This allows them to interpret the imaging as they would normally, whilst also benefit from hearing the clinical context and the management plan.

Examination delivery has fundamentally been altered due to the Covid-19 pandemic. Multiple exams were cancelled during the pandemic, namely the Spring sittings of all the examinations.^12^ Subsequently, a remote delivery system was introduced. New examination centres were chosen to accommodate for social distancing requirements. Face coverings were venue dependent except for the oral component of the FRCR 2B examination.^12^ Logistically for examiners, this may allow more flexibility to participate in exam delivery despite clinical commitments, given that consultants may not need to travel to deliver the exam. Furthermore, having examination papers in a computerised format may improve result turnover. Whilst this allows trainees who are at critical points in their training to continue progression, there are challenges. Ensuring robust internet connectivity and excellent image quality for what is already a time sensitive and high-pressure situation, is difficult particularly for the oral component of the 2B examination. Additionally, this introduces new challenges into the appeals process and how to take technical issues into account.

Conferences have also been delivered online during the pandemic. Organisations have been able to deliver the majority of the content that would been delivered in person, via an online platform of their choosing. The largest cost when organising a conference is the venue fee,^13^ with the catering also contributing a significant burden to the finances. Online conferences can be delivered without incurring either of these costs,^14^ and the majority of organisations are passing on these savings to potential attendees.

Whilst online conferences do have benefits for both attendees and organisers, there are some notable drawbacks. Firstly, the lack of an in-person event essentially precludes networking. Unexpectedly meeting people over coffee or food is often how new and valuable connections are made, and this opportunity is lost with an online conference. Online conferences are less attractive to many sponsors as it is harder to attract the attention of an attendee when the conference is online. However, there are new virtual solutions which try to increase networking and informal discussions such as Remo, a platform that has virtual discussion tables to allow mingling of attendees and sponsors.^15^

Online conference teaching can be easily recorded, subject to the consent of the teacher and trainee. Teaching cohorts of radiology trainees typically involve learners of different stages of training and different needs. Recorded content allows individuals to pause and replay content to suit their specific learning needs. Learners can even view content at faster playback rates (*e.g.* at 1.5 or 2 times the normal rate). This is useful for learners who want to revisit a topic they are already familiar with, but require a refresher. The user-directed malleability of teaching in this manner is a benefit specific to recorded content.^16^

Aside from structured teaching, radiology trainees also learn through reporting studies and reviewing cases with senior radiologists. During a pandemic, the threshold for performing an imaging examination is increased, limiting less urgent studies, and therefore reducing patient and staff cross-contamination. This has the knock-on effect of decreasing training opportunities for radiology registrars.^3^

Maintaining interventional radiology training, given its practical nature involving direct patient contact, is particularly problematic during a pandemic.^17^ The most efficient and safe practice would be to limit the number of interventionalists required for a procedure, and that would typically mean that the interventional trainees are excluded from the room. Trying to deliver or supplement interventional training by online education or by the use of simulation models, is an option,^17^ however, it is not an adequate substitute for clinical practice.

## Future directions

Social distancing continues to be necessary and as such, finding sustainable methods to allow radiology teaching and training to continue are essential. However, virtual delivery of teaching comes with many advantages and thus many of these adaptations may be here to stay. We believe radiology training may adopt a hybrid model, involving both a virtual and in-person component.

The RCR have mentioned the creation of a national or regional digital archive of images.^18^ This would allow trainees to access a much wider range of pathology from a variety of hospitals, thereby providing a more equal, homogeneous learning experience for trainees. A central database, curated by recognised experts, is also a way to ensure accuracy of the teaching materials.

The number of virtual resources has been rapidly increasing. Everlight Radiology have introduced EverLearning Webinars,^19^ Radiology Seminars^20^ also have their online series, and we believe many more will follow suit. These could be incorporated into local curricula for teaching certain topics. This would lead to a more efficient use of teacher’s time. Many introductory webinars could be used during an induction programme for incoming ST1 radiologists to prepare them with basic radiology related skills.

For conferences, recent data from the British Institute of Radiology survey^21^ demonstrate 77% of respondents from 580 respondents were more likely to engage with online material compared to pre-Covid. 72% mentioned that once it was safe to do so, they were likely to engage in a mixture of virtual and in person events, with only 10% stating they would solely prefer in person events. From a trainee perspective, from a modest sample of 13 trainees, 85% said they would be more likely to engage in online learning resource in general and 63% would be happy to engage in a mixture of in-person and virtual events, with 15% stating they would prefer virtual only events. These data are promising for the future of virtual teaching opportunities and their incorporation into radiology training.

Examination delivery has been revised and may stay that way. The FRCR 2B exams were delivered remotely, with candidates taking the exam over a virtual platform in a specific testing centre with an invigilator. Moreover, the RCR is exploring how to deliver the written examinations (FRCR Part 1 and Part 2A) so that candidates may take them in a location of their choice, including their own home.^12^ This may have impacts for international training, perhaps opening up the examinations to a wider audience. Unfortunately, no examiner report exists nor any survey of examinees for the first virtual sitting of the FRCR 2B exam, and thus it is difficult to ascertain how those involved found the experience. Anecdotally however, some trainees did experience technical difficulties during the exam but the majority found the format acceptable.

The aftermath of the pandemic will continue to affect trainee progression, especially for trainees who have missed training time (due to shielding, redeployment, sickness, or self-isolation). Health Education England (HEE) have introduced a self-assessment form prior to their Annual Review of Competency Progression (ARCP), to allow trainees to identify any issues locally to their educational supervisor. In addition, they have allocated funding to support trainees who have had an absence from training. Trainees can apply for funding to support additional courses or supernumerary clinical activities. Furthermore, there have been curriculum amendments for trainees with new Covid-19 specific ARCP outcomes to account for missing competencies trainees may not have achieved due to the pandemic and to allow training schemes to address any specific issues.

Lastly, the pandemic has had a significant impact on mental health, with many trainees having encountered difficult personal and professional situations. A recent BMA survey of over 7000 doctors has found of 41% suffering with mental health issues and 29% stating this had worsened during the pandemic. Moreover, the BMA’s well-being support services have seen a 40% increase during the first wave of the pandemic.^22^ A French study has found similar results from a survey of French radiologists.^23^ In response, the RCR been promoting more well-being resources and have hosted some well-being seminars. Given that trainee well-being has become a more prominent issue, we believe there will be greater emphasis on mental health during radiology education and training.

## Conclusion

The coronavirus pandemic has required a new approach to teaching radiology trainees. The modifications that have been necessitated by the pandemic certainly have their drawbacks, but there are also notable benefits. Some of the innovations and changes in cultural practice that have occurred during the pandemic will doubtless remain, as we move forward into the post-pandemic world. However, the precise balance of in-person *vs* online education remains to be seen.
